# Progress in Fevers

**Published:** 1904-02-20

**Authors:** 


### PROGRESS IN FEVERS.

**Trypanosomiasis.** — Since the publication by Dutton and Ford of the first recorded case of this disease several other examples of human infection with an organism resembling that found in cattle suffering from the bite of the tsetse fly have been brought forward. The symptoms consist in irregular remittent or intermittent fever of a malarial type, headache, wasting, and weakness, œdema, erythema, and splenic enlargement. Manson and Daniels[22] record a case under their care at the Seamen's Hospital. In this patient, a lady, resident for fifteen months on the Upper Congo, trypanosomes were repeatedly found in the blood. The pulse was always rapid and feeble, the temperature irregular and moderate, rarely very high. The spleen extended at one time forwards to the umbilicus and downwards to the iliac crest. Œdema, never very excessive, involved chiefly the face and lower part of back, where also the erythema, which was marked but patchy and variable in character, was chiefly situated, although it also involved the limbs. The eyes showed patches of choroidal atrophy. Weakness and general ill-health were very pronounced. The red blood cells averaged about 3,000,000 or more; white cells were slightly decreased. The patient's blood after admixture with 5 per cent. sodium citrate solution was injected experimentally into rats, mice, guinea-pigs, monkeys, and also into a pony, a rabbit, and a dog, but with entirely negative results in all cases. Arsenic, hypodermically injected in the form of arrhenal, cacodylate of sodium, or arsenate of iron, was pushed to the limit of prudence, but without benefit. Methylene blue was also tried. As the case appeared grave and resistant to other treatment, injections of animal serum were resorted to, that of the horse being selected. The injections were, however, followed by rise of temperature, rapid fall in blood count, and, on one or two occasions, by such alarming depression and collapse that they had to be discontinued. This treatment was based on the observation of Larceau that mice infected with the trypanosoma of nagana, a disease almost invariably fatal to them, recovered when injected with human blood serum. Man being immune as regards nagana, and the horse having been proved to be immune to the trypanosoma of this patient, it was hoped that an analogous good result might be obtained. Recovery from the effects of the serum was rapid. This patient subsequently[23] recovered for a few months a fair amount of health. Fever and weakness, however, speedily returned, and later symptoms of drowsiness. When seen at her home in Bristol in October by Sir Patrick Manson and Dr. Low the patient was extremely weak and wasted, the rapid pulse and ringed erythema previously observed were still present, and trypanosomes were more numerous in the blood. The patient could be roused to converse, but in the intervals of conversation she appeared to sleep. Twitching at the left angle of the mouth was observed. Later the patient became gradually more drowsy, and conversation was confined to monosyllables. Control of the sphincters was lost, spasm of one arm occurred, bed sores formed, and she died comatose on November 26th, 1903, rather more than two years after the beginning of the illness. At the necropsy, made by Dr. Neild, of University College, Bristol, signs of chronic meningo-encephalitis, viz., injection of vessels and milkiness of the pia arachnoid were found. Subsequently microscopical examination of sections of the brain showed the extensive peri-vascular small mononuclear infiltration characteristic of sleeping sickness. This is the first well-authenticated case of sleeping sickness recorded in a European, and its association with the trypanosoma is regarded as of great etiological significance. The case was brought to Manson's notice by Dr. Habershon,[24] who, in treating the patient at first for malaria, recognised its great obscurity. Leishman thinks that trypanosomiasis may not improbably be found to have a wide distribution in India and other tropical countries, and that some cases of "Dum-Dum fever" may be really examples of this disease.[25] Dum-Dum is a notoriously unhealthy military cantonment about seven miles from Calcutta, from which obscure cases of cachexia and remittent fever are frequently invalided home to Netley. One such case, which occurred in 1900, and was also the subject of chronic dysentery, is described. Post-mortem the spleen was found greatly congested, weighing 2 lbs. 7 ozs. Microscopic examinations of smear preparations of the pulp showed the presence scattered between the splenic and blood cells of enormous quantities of small round or oval bodies possessing a large quantity of chromatin. Similar bodies were not found, however, in the spleen pulp of two other fatal cases of Dum-Dum fever of a similar type. In these cases the spleen was less enlarged. No explanation of the bodies was then found, but recently the same observer found almost identical chromatin bodies in the blood and internal organs of a white rat dead of nagana, the trypanosoma of tsetse-fly disease. They were considered to be the degenerated remains, the macro- and micro nuclei, of the adult trypanosomes, all the stages of the degenerative changes being capable of being traced. The staining reactions of the bodies found in each case were similar, and also their shape. Those found in the human spleen were somewhat smaller, a fact which might be explained by their representing a different species. The occurrence of the bodies is held, at any rate, to suggest the strong possibility of this case of Dum-Dum fever having been infected with trypanosomes. The fact that trypanosomiasis, although possibly of not infrequent occurrence in the tropics, has hitherto eluded observation, may be explained, Leishman thinks, by the rapid disintegration of the parasites which appears to take place soon after the death of the host, and by the fact that in animals which are resistent to infection by this parasite, the organisms appear to be restricted to the internal organs, and are not found in the peripheral circulation. More recently Captain Donovan[26] has described precisely similar bodies observed in post-mortem smears from the spleens of three cases of supposed chronic malaria who died in Madras. He also found the same bodies in two cases in the splenic blood withdrawn during life. Ross confirms the careful accuracy of Leishman's descriptions, but doubts whether the bodies observed by him and subsequently by Donovan are really involution forms of trypanosomes found in post-mortem spleens, since no flagella or other traces of dead trypanosomes were found. Further, some of the bodies appear to be intracorpuscular, and were found by Donovan in the splenic blood of living subjects. Ross thinks the organism described is a new one, and states that the charts of Donovan's cases in which the organism was found suggest kala-azar to his mind. Leishman[27] admits that some of the bodies appear to be actually within the red blood-cells in Donovan's slides, and that this intracorpuscular position is against his view of their being trypanosomes degenerated post-mortem, but does not yet regard his theory as having been disproved by Ross. C. J. Baker[28] found trypanosomes in the blood of three men, natives of Uganda, who had never been outside the Protectorate. The cases were all slight, and recovered rapidly. This country is said to be free from tsetse-fly disease among cattle, but a member of the group has been found.

[22] Brit. Med. Jour., May 30. [23] Ibid., Dec. 5. [24] Ibid., Dec. 12, p. 1565. [25] Ibid., May 30. [26] Ibid., Nov. 14, p. 1261. [27] Ibid., Nov. 21, p. 1376. [28] Ibid., May 30.

(*To be continued.*)

